# The effect of different treatment strategies on the right heart dysfunction during follow-up in the patients with acute pulmonary thromboembolism of intermediate-risk

**DOI:** 10.3389/fcvm.2025.1641886

**Published:** 2025-09-29

**Authors:** Yin Wang, Bo Li, Chunyan Rong, Ming Lu, Weihua Zhang

**Affiliations:** Department of Cardiovascular Medicine, The First Hospital of Jilin University, Changchun, China

**Keywords:** acute pulmonary embolism, pulmonary artery thrombus aspiration, anticoagulation therapy, thrombolysis therapy, right heart dysfunction, catheter-directed thrombectomy

## Abstract

**Background:**

Acute pulmonary embolism (APE) is a disease with a high incidence and mortality rate. Currently, the preferred treatment methods for low - risk and high - risk of APE have been clearly defined, but there is still controversy regarding the optimal treatment for the intermediate - risk of APE. Patients with intermediate-risk APE have a relatively high thrombus burden, which can cause right heart function impairment during the follow-up period. It is still uncertain whether reperfusion therapy has an impact on right heart function during the follow-up period.

**Objectives:**

This study aims to evaluate the impact of reperfusion therapy on right heart function during the follow-up period in intermediate-risk APE patients by comparing the effects of different treatment strategies on right heart dysfunction (RHD) at 3 months.

**Methods:**

This study retrospectively included 216 patients who met the inclusion and exclusion criteria of this study. According to the treatment methods used, the patients were divided into the thrombolysis group, the catheter-directed thrombectomy group and the anticoagulation group.

**Results:**

In this study, the median follow - up time was 4.2 (3.1, 5.4) months. Among the patients with intermediate-risk APE, the incidence of RHD in the catheter-directed thrombectomy group was lower than that in the anticoagulation group (*P* < 0.05). Among the patients with intermediate-high-risk APE, the incidence of RHD events after 3 months in both the thrombolysis group and the catheter-directed thrombectomy group was lower than that in the anticoagulation group (*P* < 0.05). Catheter-directed thrombectomy reduced the probability of RHD at 3 months to 0.12 of that with anticoagulation therapy alone (OR = 0.12, 95% CI: 0.015–0.994, *P* = 0.049).

**Conclusion:**

In patients with intermediate-risk APE, reperfusion therapy may reduce the occurrence of RHD impairment events after 3 months and improve the prognosis of patients.

## 1 Introduction

Acute pulmonary embolism (APE) is a disease with high incidence and high mortality. It is the third leading cause of death among cardiovascular diseases after myocardial infarction and stroke (1–3). Epidemiological studies in Western countries have shown that the annual incidence of APE in the general population is approximately 39–115 per 100,000 (2). According to the 2019 ESC Guidelines for the diagnosis and management of APE (2), defining intermediate - risk pulmonary embolism (PE) poses a serious challenge. Although patients with intermediate - risk APE have stable hemodynamics, at least one of the cardiac biomarkers and right ventricular dysfunction is abnormal, and both are independently associated with an increased risk of mortality. According to the latest domestic and international guidelines (1, 2), direct thrombolysis is not recommended for patients with intermediate - risk APE. Only when there is hemodynamic impairment, rescue thrombolysis can be performed. If there are contraindications to thrombolysis, surgical thrombectomy or catheter - based interventional therapy can be used as an alternative to rescue thrombolysis. Anticoagulation therapy is the fundamental treatment for APE. Reperfusion treatment, including pharmacological thrombolysis and interventional therapy, can rapidly reduce the cardiac load and relieve hemodynamic pressure. The benefits of pulmonary reperfusion treatment in intermediate - risk APE remain unclear, and the results of multiple randomized trials vary. In this study, we compared the incidence of RHD among intermediate - risk APE patients who received different treatment strategies after 3 months, aiming to evaluate the impact of reperfusion treatment on right heart function during the follow - up period in patients with intermediate - risk APE.

## 2 Materials and methods

### 2.1 General information of patients

**Figure 1:**
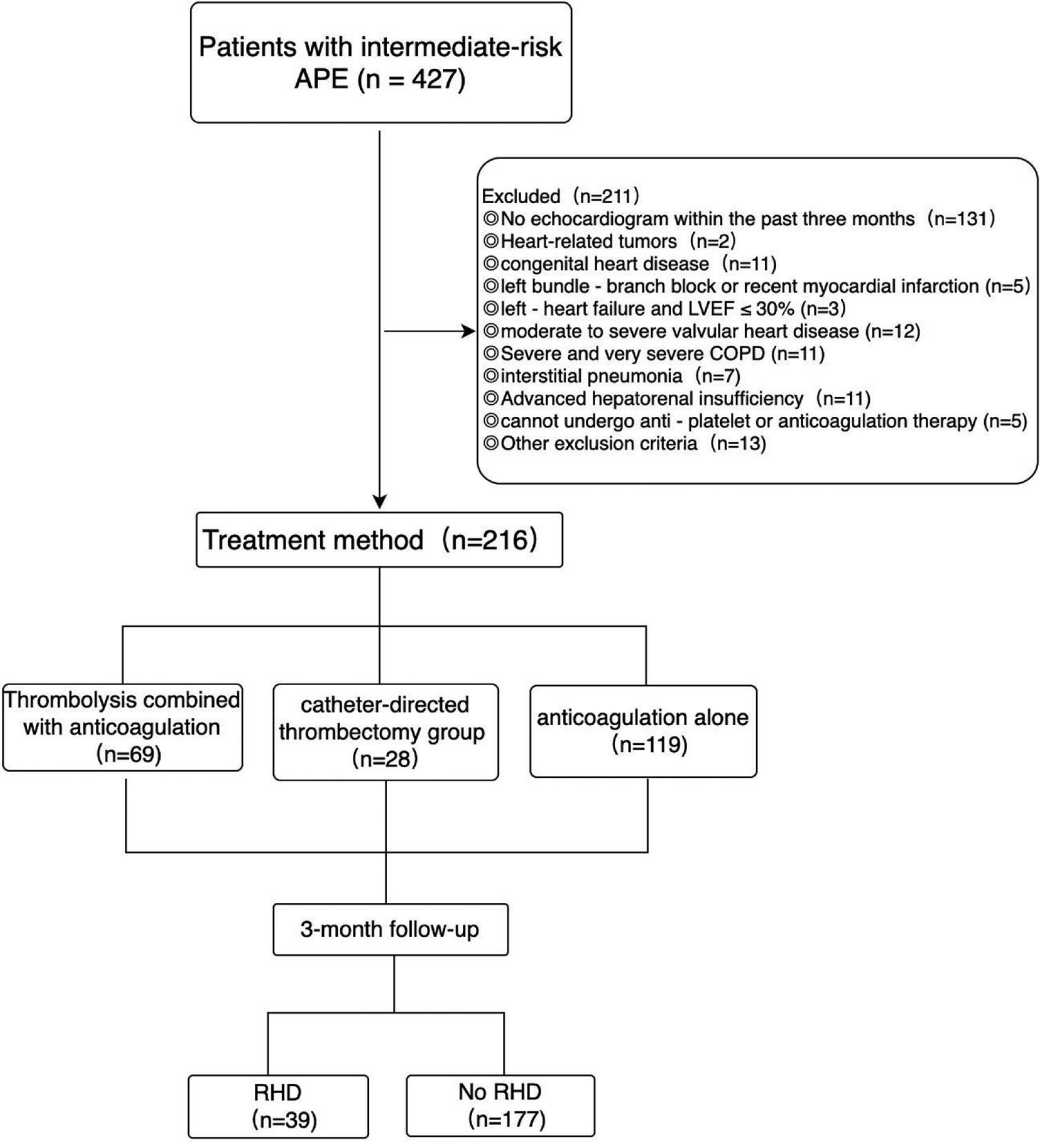
Enrollment and follow - up of patients with APE in the intermediate - risk group.

A total of 216 patients who were diagnosed with APE in the intermediate - risk group and had complete clinical data were retrospectively enrolled. These patients were hospitalized in the Department of Cardiology at the First Hospital of Jilin University from August 2018 to April 2024. Among them, 69 patients received thrombolysis combined with anticoagulation therapy, 28 patients underwent catheter-directed thrombectomy combined with anticoagulation therapy, and 119 patients were treated with anticoagulation therapy alone, the patients were followed up for at least 3 months, with a median follow - up time of 4.2 months (Figure 1). This study was conducted in strict accordance with the revised Declaration of Helsinki. The research protocol was meticulously reviewed and approved by the Ethics Committee of the First Hospital of Jilin University (Ethics Approval Number: 2025 - 048). Moreover, written informed consent was obtained from all patients, ensuring that they were fully aware of the study details and voluntarily participated in the research. In this study, the diagnosis of APE in the intermediate - risk group should first meet the diagnostic criteria and risk stratification standards outlined in the 2019 ESC Guidelines for the diagnosis and management of APE. In addition, patients were excluded according to the following criteria: patients with contraindications for pulmonary artery catheterization, such as prostheses or vegetations of the tricuspid or pulmonary valves, left bundle - branch block, or recent myocardial infarction; patients with a history of left - heart failure and a left ventricular ejection fraction (LVEF) ≤30%; patients with congenital heart disease, moderate to severe valvular heart disease, cardiomyopathy, cardiac amyloidosis, fabry disease, or constrictive pericarditis; Severe and very severe chronic obstructive pulmonary disease (COPD, with FEV₁/FVC still <70% and FEV₁ percentage of the predicted value <50%), interstitial pneumonia, or active pulmonary tuberculosis; patients with preoperative renal function test results indicating serum creatinine >1.8 mg/dl or 159 μmol/L; preoperative liver function score indicates: Child - Pugh class C; patients who cannot undergo anti - platelet or anticoagulation therapy (Platelets <50 × 10^9^/L or INR >3); patients who have undergone open cardiovascular or pulmonary surgery within 7 days before the operation; Heart-related tumors, systemic infectious diseases, sepsis, the patient's general condition not being able to tolerate surgery, and the expected lifespan being less than 3 months; pregnant and lactating women; patients who are participating in other drug or medical device clinical trials and those with incomplete clinical data.

### 2.2 Clinical features and laboratory examination

A retrospective analysis was carried out on the clinical data of all the enrolled patients. General baseline data of the patients were collected, including blood gas analysis, complete blood count, liver function, biochemical parameters, troponin, B - type natriuretic peptide (BNP), and D - dimer levels upon admission. Additionally, echocardiographic parameters were gathered both at the time of admission and after a three - month follow - up period, such as left ventricular ejection fraction (LVEF), left ventricular dimension (LV), right ventricular dimension (RV), right atrial dimension (RA), right - to - left ventricular dimension ratio (RV/LV), tricuspid annular plane systolic excursion (TAPSE), tricuspid regurgitation peak gradient (TRPG), and systolic pulmonary artery pressure (SPAP).

### 2.3 Echocardiography

All patients underwent echocardiography both at the time of admission and after three months to assess right heart function and pulmonary artery pressure. These echocardiographic examinations were performed by senior cardiac ultrasonographers. Based on the echocardiographic findings, patients were divided into the RHD group and the non - RHD group. The criteria for the RHD group included the presence of a RV end - diastolic diameter >28 mm, a RA superior - inferior diameter >50 mm or a RA left - right diameter >40 mm, a SPAP >40 mmHg, or TRPG >36 mmHg, indicating right heart enlargement and/or elevated pulmonary artery pressure.

### 2.4 Imaging evaluation of APTE

Computed tomography pulmonary angiography (CTPA) was employed to comprehensively assess the pathological conditions of the pulmonary arteries. The CTPA images were meticulously reviewed by two highly experienced radiologists. The involvement status of the main pulmonary artery, left and right pulmonary arteries, and pulmonary artery branches was carefully recorded. Additionally, typical imaging manifestations were documented, including low - density filling defects, stenosis, and occlusion of the pulmonary arteries, to facilitate further in - depth analysis.

### 2.5 Treatment

The treatment of APTE adhered to the drugs and dosages recommended by the guidelines. Patients in the thrombolysis group initially received alteplase (either 50 mg or 0.6 mg/kg, administered via a 2 - hour pump infusion). Subsequently, they were subjected to standardized anticoagulation therapy. For the catheter-directed thrombectomy group, domestic pulmonary artery thrombus aspiration catheters and thrombus retrieval stents were utilized. The catheters and stents were sized between 16 and 20 Fr and were manufactured by Chenxing Medical Devices Co., Ltd. and Shanghai Rongmai Medical Technology Co., Ltd. In the anticoagulation group, patients were given enoxaparin (either 100 U/kg once every 12 h or 1.0 mg/kg once every 12 h) or dalteparin sodium (either 100 U/kg once every 12 h or 200 U/kg once a day). After discharge, all patients were switched to oral rivaroxaban therapy. They initially took 15 mg twice daily. After three weeks, the dosage was adjusted to 20 mg once daily. Meanwhile, during the treatment process, when a patient experienced intracranial hemorrhage, gastrointestinal hemorrhage, pericardial hemorrhage, a hemoglobin drop of ≥20 g/L, or required a transfusion of ≥2 units of red blood cells, it was defined as a major bleeding event.

### 2.6 Statistical analysis

Statistical analysis was conducted using SPSS 26 software. For measurement data that conformed to a normal distribution, they were presented as the mean ± standard deviation (x ± s). The one - way analysis of variance (ANOVA) was employed for comparisons between groups. For *post hoc* tests, the Tukey's Honestly Significant Difference (HSD) method was selected based on the homogeneity of variances. In cases where the data did not follow a normal distribution, they were described as the median (inter - quartile range) [M (P25–P75)]. The differences between groups were analyzed using the Kruskal–Wallis *H*-test, and pairwise comparisons were performed using the Mann–Whitney *U*-test with Bonferroni correction. Count data were presented as the number of cases (percentage) [*n* (%)]. For analyzing the differences between groups, the chi - square (χ^2^) test or Fisher's exact probability test was selected according to the frequency conditions. Univariate Logistic regression analysis was first used to screen variables. Variables with *P* < 0.05 were further subjected to multivariate Logistic regression analysis to adjust for confounding factors. The odds ratio (OR) and its 95% confidence interval (95%CI) were calculated. A *P*-value <0.05 was considered to indicate a statistically significant difference. For individual missing indicators, the mean substitution method was used to handle the missing data in SPSS software (Among the included data, 15 patients had no TRPG data, 21 patients had no TAPSE data, and 23 patients had no SPAP data).

## 3 Results

### 3.1 Characteristics of patients

In the general data, there were 69 patients in the thrombolysis group. The median age was 61 years (IQR 55–68), with 35 (50.7%) being male patients and 55 (79.7%) classified as intermediate-high-risk APE. In the catheter-directed thrombectomy group, there were 28 patients. The median age was 64 years (IQR 55–70), including 10 (35.7%) male patients and 22 (77.6%) intermediate-high-risk APE. In the anticoagulation group, there were 119 patients. The median age was 65 years (IQR 59–70), with 57 (47.9%) male patients and 90 (75.6%) intermediate-high-risk APE (Table 1). There were no statistically significant differences in age, gender, risk stratification, heart rate (HR), respiratory rate (RR), systolic blood pressure (SBP), diastolic blood pressure (DBP), partial pressure of oxygen (PO_2_), oxygen saturation (SaO₂), hemoglobin (HB), platelet count (PLT), aspartate aminotransferase (AST), alanine aminotransferase (ALT), creatinine (Cr), RV, RA, RV/LV, TRPG, TAPSE, SPAP, presence of elevated cardiac troponin I (cTnI), presence of elevated B - type natriuretic peptide/N-terminal pro-B-type natriuretic peptide (BNP/NT-proBNP), involvement of the main trunk on CTPA, presence of cardiac dysfunction, history of surgery or trauma within 1 month, comorbidity of hypertension, diabetes, tumor, or deep vein thrombosis (DVT) among the three treatment modalities (*P* > 0.05) (Table 1).

**Table 1:** Comparison of baseline data at different treatment methods during admission.

Inspection items	Thrombolysis group (*n* = 69)	Catheter-directed thrombectomy group (*n* = 28)	Anticoagulation group (*n* = 119)	χ^2^/F/H	*P*
Age (years)	61 (55, 68)	64 (55, 70)	65 (59, 70)	1.592	0.206
Gender (*n*, %)					
Male	35 (50.7)	10 (35.7)	57 (47.9)	1.849	0.397
Female	34 (49.3)	18 (64.3)	62 (52.1)		
Risk stratification (*n*, %)					
Intermediate-high-risk	55 (79.7)	22 (78.6)	90 (75.6)	0.443	0.801
Intermediate-low-risk	14 (20.3)	6 (21.4)	29 (24.4)		
HR (times/minutes)	90 (78, 100)	88 (80, 100)	90 (78, 101.5)	0.260	0.771
RR (times/minutes)	19 (18, 20)	18 (18, 19.75)	18 (18, 20)	0.207	0.813
SBP (mmHg)	125.67 ± 17.07	124.14 ± 11.67	130 ± 12.79	2.504	0.089
DBP (mmHg)	77 (68, 86)	78 (70, 90)	80 (71, 90)	1.846	0.160
P0_2_ (mmHg)	66 (60, 70)	70 (65, 73)	69 (61, 80)	2.833	0.061
SaO_2_ (%)	94 (91, 96)	95 (93, 96)	96 (93, 98)	1.884	0.155
HB (g/L)	142.2 ± 17.60	138.89 ± 22.25	138.64 ± 23.45	0.624	0.537
PLT (×10^9^/L)	195 (158, 215)	210 (173, 228)	203 (162, 240)	1.727	0.180
AST (U/L)	25 (19.2, 31.5)	24.8 (18.7, 32.33)	23.6 (19.35, 34.2)	0.586	0.558
ALT (U/L)	23.4 (15.6, 36.1)	26.7 (19.08, 44.45)	21.4 (14.3, 35.2)	0.375	0.688
Cr (umol/L)	68.6 (57.3, 84.4)	65 (53.65, 74.63)	68.9 (57.9, 89.6)	1.645	0.195
cTnI increase (*n*,%)	59 (85.5)	23 (82.1)	86 (72.3)	4.783	0.091
BNP/NT-proBNP increase (*n*, %)	61 (88.4)	25 (89.3)	106 (89.1)	0.025	0.988
RV (mm)	29 (25.5, 31.5)	29 (27.25, 30)	27 (25, 30)	1.115	0.330
RA (mm)	47.5 (44, 51)	47.5 (46.5, 49)	47 (43.62, 50.5)	0.098	0.907
RV/LV	0.65 (0.58, 0.78)	0.68 (0.59, 0.76)	0.64 (0.55, 0.83)	0.054	0.948
TRPG (mmHg)	41 (30, 51)	46.5 (33, 56)	42 (30, 55)	0.604	0.547
TAPSE (mm)	15.42 ± 3.717	16.47 ± 2.416	15.88 ± 3.660	0.422	0.657
SPAP (mmHg)	56 (44, 77)	59 (47, 67)	56 (49, 67)	0.620	0.541
Baseline medical history (*n*, %)					
CTPA main burden	56 (81.2)	21 (75)	90 (75.6)	0.860	0.651
RHD	53 (76.8)	24 (85.7)	90 (75.6)	1.329	0.515
1 month's surgical trauma history	2 (2.9)	3 (10.7)	9 (7.6)	–	0.254
Hypertension	15 (21.7)	11 (39.3)	44 (37)	5.323	0.070
Diabetes	8 (11.6)	5 (17.9)	21 (17.6)	1.315	0.518
Tumors	5 (7.2)	1 (3.6)	9 (7.6)	–	0.866
DVT	69 (100.0)	27 (96.4)	115 (96.6)	–	0.319

HR, heart rate; SBP, systolic blood pressure; DBP, diastolic blood pressure; P02, partial pressure of oxygen; SaO2, arterial oxygen saturation; HB, hemoglobin; PLT, platelets; AST, aspartate aminotransferase; ALT, alanine aminotransferase; Cr, creatinine; cTnI, cardiac troponin I; BNP/NT-proBNP, B - type natriuretic peptide/N-terminal pro-B-type natriuretic peptide; RV, right ventricle; RA, right atrium; RV/LV, right ventricle/left ventricle; TRPG, tricuspid regurgitant pressure gradient; TAPSE, tricuspid annular plane systolic excursion; SPAP, systolic pulmonary artery pressure; CTPA, computed tomography pulmonary angiography; RHD, right heart dysfunction; DVT, deep vein thrombosis.

With a *P*-value <0.05 and indicates that there is a statistical difference.

### 3.2 Comparison of RHD events in the follow-up period

A follow - up was conducted on 216 patients with intermediate - risk APE after 3 months. Among them, 39 cases (18.06%) developed RHD. In the thrombolysis group, 10 cases (14.5%) developed RHD; in the catheter-directed thrombectomy group, 1 case (3.6%) developed RHD; and in the anticoagulation group, 28 cases (23.5%) developed RHD. There were statistically significant differences in the incidence of RHD among the three groups during the follow - up period (*P* < 0.05). The incidence of RHD in the catheter-directed thrombectomy group during the follow - up period was lower than that in the anticoagulation group, with a statistically significant difference (*P* < 0.05). There were no statistically significant differences in the incidence of RHD during the follow - up period between the thrombolysis group and the catheter-directed thrombectomy group, as well as between the thrombolysis group and the anticoagulation group (*P* > 0.05) (Table 2). In this study, none of the 216 included patients experienced major bleeding events or died. Moreover, no surgery - related complications occurred in the patients who underwent catheter-directed thrombectomy treatment.

**Table 2:** The impact of different treatment methods on RHD in the follow-up period.

RHD (*n*, %)	Thrombolysis group	Catheter-directed thrombectomy group	Anticoagulation group	χ^2^	*P*
Intermediate - risk APE	10 (14.5)	1 (3.6)^c^	28 (23.5)	6.972	0.031^a^
Intermediate - high - risk APE	8 (14.5)^b^	1 (4.5)^c^	27 (30.0)	–	0.009^a^
Intermediate - low - risk APE	2 (14.3)	0 (0.0)	1 (3.4)	–	0.493

^a^ Indicates a statistically significant difference among the three groups.

^b^ Indicates a statistically significant difference between the thrombolysis group and the anticoagulation group.

c Indicates a statistically significant difference between the intervention group and the anticoagulation group (*P* < 0.05).

A 3 - month follow - up was carried out on 167 patients with intermediate - high - risk APE. There were statistically significant differences in the incidence of RHD among the three groups during the follow - up period (*P* < 0.05). The occurrence of RHD events after 3 months in both the thrombolysis group and the catheter-directed thrombectomy group was lower than that in the anticoagulation group, and the differences were statistically significant (*P* < 0.05). However, there was no statistically significant difference in the incidence of RHD between the thrombolysis group and the catheter-directed thrombectomy group during the follow - up period (*P* > 0.05) (Table 2).

A three - month follow - up was conducted on 49 patients with intermediate - low - risk APE. There were no statistically significant differences in the incidence of RHD among the three groups during the follow - up period (*P* > 0.05) (Table 2).

### 3.3 Factors affecting RHD in the follow-up period

In this study, univariate logistic regression analysis was employed to investigate the influencing factors of RHD during the follow - up period among patients with APE in the intermediate - risk group. Subsequently, the indicators with a *P* - value less than 0.05 in the univariate analysis and those with clinical significance were included in the multivariate logistic regression analysis. Univariate logistic regression analysis revealed thatcatheter-directed thrombectomy approaches reduced the likelihood of RV dysfunction at 3 months by 8.3 amount (OR = 0.120, 95% CI: 0.016–0.926, *P* = 0.042). Meanwhile, univariate logistic regression analysis indicated that hazard stratification, baseline RHD, RV, RA, RV/LV, TRPG, and SPAP were all correlated with the occurrence of RHD after 3 months (*P* < 0.05) (Table 3). Since there was a linear correlation between TRPG and SPAP, only TRPG was included in the multivariate logistic regression analysis. The multivariate logistic regression analysis indicated that Catheter-directed thrombectomy reduced the probability of RHD at 3 months to 0.12 of that with anticoagulation therapy alone (OR = 0.120, 95% CI: 0.015–0.994, *P* = 0.049). RA value was an independent risk factor for the development of RHD after 3 months (OR = 1.167, 95% CI: 1.045–1.303, *P* = 0.006) (Table 4).

**Table 3:** 3-month follow-up single-factor logistic regression analysis of RHD.

Statistic variables	Groups	B	Waldχ^2^	*P*	OR	95% CI
Treatment method	1				1.000	
	2	−0.596	2.173	0.140	0.551	0.249–1.217
	3	−2.117	4.136	0.042*	0.120	0.016–0.926
Age (years)		0.003	0.023	0.880	1.003	0.969–1.038
Gender (male)		0.451	1.597	0.206	1.569	0.78–3.156
Risk stratification (intermediate-high-risk)		1.438	5.298	0.021*	4.214	1.238–14.341
HR (times/minutes)		0.000	0.000	0.991	1.000	0.978–1.023
RR (times/minutes)		−0.021	0.237	0.627	0.980	0.901–1.065
SBP (mmHg)		0.006	0.203	0.653	1.006	0.982–1.03
DBP (mmHg)		0.025	3.317	0.069	1.025	0.998–1.054
P02 (mmHg)		0.012	0.619	0.431	1.012	0.982–1.043
SaO2 (%)		0.033	0.559	0.455	1.034	0.947–1.128
HB (g/L)		0.008	0.992	0.319	1.008	0.992–1.025
PLT (×10^9^/L)		0.001	0.068	0.795	1.001	0.995–1.006
AST (U/L)		0.006	1.740	0.187	1.006	0.997–1.014
ALT (U/L)		0.003	1.087	0.297	1.003	0.998–1.008
CR (umol/L)		0.004	0.253	0.615	1.004	0.988–1.021
cTnI increase *n* (%)		1.063	3.658	0.056	2.895	0.974–8.603
BNP/Pro-BNP increase *n* (%)		19.836	0.000	0.998		
RV (mm)		0.103	7.762	0.005*	1.109	1.031–1.192
RA (mm)		0.171	17.857	0.000*	1.186	1.096–1.284
RV/LV		2.131	5.496	0.019*	8.425	1.418–50.044
TRPG (mmHg)		0.046	18.735	0.000*	1.047	1.026–1.07
TAPSE (mm)		−0.057	0.520	0.471	0.945	0.81–1.102
sPAP (mmHg)		0.060	10.709	0.001*	1.062	1.024–1.101
Concomplication *n* (%)						
RHD		1.900	6.495	0.011*	6.688	1.551–28.842
CTPA main burden		20.014	0.000	0.997	1.000	0
1 month's surgical trauma history		−1.103	1.096	0.295	0.332	0.042–2.616
Hypertension		0.241	0.383	0.536	1.273	0.593–2.733
Diabetes		0.287	0.304	0.581	1.332	0.481–3.694
Tumors		−19.779	0.000	0.998	0.000	0
DVT		−0.129	0.013	0.909	0.879	0.095–8.084

“1” means anticoagulation treatment; “2” means thrombolysis treatment; “3” means catheter-directed thrombectomy treatment.

* With a *P*-value <0.05 and indicates that there is a statistical difference.

**Table 4:** 3-month follow-up multi-factor logistic regression analysis with RHD.

Statistic variables	Groups	B	Wald*χ*^2^	*P*	OR	95% CI
Treatment method	1		4.932	0.085		
	2		1.749	0.186	0.524	0.201–1.366
	3	−2.118	3.863	0.049*	0.120	0.015–0.994
Risk stratification (intermediate-high-risk)		−0.583	0.446	0.504	0.558	0.101–3.092
RV (mm)		−0.096	1.391	0.238	0.909	0.775–1.065
RA (mm)		0.155	7.558	0.006*	1.167	1.045–1.303
RV/LV		1.442	0.772	0.380	4.229	0.17–105.408
TRPG (mmHg)		0.025	3.677	0.055	1.025	0.999–1.052
RHD *n* (%)		19.271	0	0.998	–	

Abbreviations as in Table 1. “1” means anticoagulation treatment; “2” means thrombolysis treatment; “3” means catheter-directed thrombectomy treatment.

* With a *P* value <0.05 and indicates that there is a statistical difference.

## 4 Discussion

The registration of FLASH (4) indicates that, despite hemodynamic stability, more than one - third of patients with intermediate - risk APE are in a state of normotensive shock with a reduced cardiac index. In the intermediate - risk group, APE can lead to death during the acute phase and events such as chronic thromboembolic pulmonary hypertension (CTEPH) and right - heart failure during the chronic phase (3). There has always been a controversy over whether patients with intermediate - risk PE can benefit from reperfusion therapy. In this study, 216 patients with intermediate - risk PE were retrospectively included. They were treated with anticoagulation alone, low - dose alteplase thrombolysis, and thrombectomy using a newly launched domestic interventional thrombectomy device respectively. The patients were followed up for more than 3 months, and it was found that reperfusion therapy could reduce the incidence of right - heart dysfunction in patients.

The study by Matusov Y et al. (5) included 113 patients at intermediate - high risk. Among them, 58 (51.3%) received anticoagulation therapy alone, 12 (10.6%) received systemic thrombolysis, and 43 (38.1%) received catheter - directed intervention. The patients were followed up for at least 6 months. It was found that regardless of the baseline RV function, compared with anticoagulation therapy alone, patients with intermediate - high - risk PE who received catheter - directed intervention or systemic thrombolysis were more likely to achieve long - term recovery of RV function. Our study design is similar to this one, and our results also suggest that reperfusion therapy is beneficial for improving right heart function. in this study, the median follow - up time was 4.2 (3.1, 5.4) months, which is relatively short. A prospective study (6) that enrolled 55 patients with intermediate-risk APE: The patients were followed up for 3 months, among those who received anticoagulation therapy, 27.27% developed right heart failure (RHF), among the patients who received thrombolytic therapy, there was no hemodynamic decompensation except for tachycardia (30%). Numerous studies (7–9) have confirmed the effectiveness and safety of interventional therapy for APE. It can reduce the occurrence of adverse events and alleviate cardiac afterload during the postoperative period and within 48 h. However, none of these studies have explored the function and status of the right heart three months after interventional therapy. In this study, domestic devices were used to perform interventional thrombus aspiration and thrombectomy on 28 patients. No adverse events occurred during the operation for any of the patients. Three months later, only one patient (3.6%) developed RHD.

Reperfusion therapy can rapidly and effectively reduce the thrombus burden and restore pulmonary circulation (10). However, the findings of quite a few studies still suggest that reperfusion therapy has no association with the occurrence of adverse events in pulmonary embolism. A meta - analysis (11) that included 33 studies with a total of 3,920 patients followed up the patients for at least 3 months. The prevalence of right ventricular dysfunction (RVD) was 0.64 (95% CI 0.42, 0.87, I^2^ = 99%). The prevalence of RVD in patients receiving thrombolytic therapy (0.17 and 0.07 at 1 - year for systemic thrombolysis and catheter - directed thrombolysis respectively) was lower than that in patients receiving anticoagulant therapy alone (0.24 at 1 - year). However, the relative risk (RR) was not statistically significant. Another meta - analysis (12) that included 26 studies with 3,671 patients and had a median follow - up time of 6 months showed that compared with the anticoagulant therapy group, the incidence of RVD was lower in the thrombolytic therapy group (odds ratio: 0.51, 95% CI: 0.24–1.13, *P* = 0.10), but the difference was not statistically significant. Golpe R et al. (13) conducted a 6 - month follow - up of 101 hemodynamically stable patients. The results showed that persistent RVD or PH after APE was not associated with long - term adverse events. However, the results of this study suggest that compared with anticoagulant therapy alone, thrombolytic therapy and catheter-directed thrombectomy therapy can reduce the incidence of RHD in patients. The reasons may be that this study is a retrospective study with a small sample size. Meanwhile, the median follow - up time was only 4.2 months, and it takes a certain period for patients’ cardiac function to recover. Therefore, whether reperfusion therapy has an impact on the long - term right heart function and status of patients with PE remains to be verified.

Domestic and international guidelines (1, 2) do not recommend direct reperfusion therapy for patients with APE in the intermediate - risk group. This is mainly because reperfusion therapy can increase the risk of bleeding. Many previous studies (14–16) have reported that intravenous thrombolysis can increase the risk of bleeding. The Pulmonary Embolism Thrombolysis Trial (PEITHO) (16) is the largest randomized controlled trial (RCT) evaluating hemodynamically stable patients. This study randomly selected 1,006 patients in the intermediate-risk group. The results showed that the administration of systemic thrombolysis (tenecteplase 30–50 mg) was beneficial for the composite endpoint of mortality or hemodynamic collapse at 7 days after randomization. In this study, the incidence of the primary endpoint events in the thrombolysis group was significantly reduced (2.6% vs. 5.6%; *P* = 0.015), but the risk of major bleeding was significantly increased (6.3% vs. 1.5%, *P* < 0.001), especially the risk of intracranial hemorrhage. However, the cohort study by Leandro Bobadilla et al. (10), which included 178 patients with intermediate - high - risk APE, showed that the rate of minor bleeding in patients who received reperfusion therapy was 7.1%, much lower than the 32% bleeding rate observed in randomized studies such as PEITHO study. Moreover, compared with patients who did not receive reperfusion therapy, the short - term mortality rate of patients who received reperfusion therapy was 10 percentage points lower (3.6% vs. 14%; OR 0.22; 95% CI 0.02–1.76; *P* = 0.1). There are discrepancies in the results reported in the literature regarding whether reperfusion therapy increases the risk of bleeding. In this study, none of the patients who received thrombolytic therapy experienced major bleeding events. This may be related to the use of low - dose thrombolytic agents. Meanwhile, the enrolled patients had fewer underlying diseases, so the corresponding bleeding risk was also lower. Since bleeding is considered a short - term effect, the bleeding risk associated with thrombolysis was not evaluated as a long - term outcome. Although the guidelines do not recommend direct thrombolytic therapy for patients with intermediate - risk PE, in actual clinical research, doctors should assess the thrombus burden and the status of cardiopulmonary dysfunction in patients with intermediate - risk APE to decide whether to administer thrombolytic therapy.

To date, a unified definition of RHD has not been established. In this study, in collaboration with the echocardiography department of our hospital, we innovatively defined RHD and summarized specific parameters to more comprehensively describe the long - term status of the right ventricle: RV, RA, TPRG, SPAP, RV/LV, TAPSE, these parameters more comprehensively represent the right ventricular systolic function, morphological changes, and pressure load. The study found that the risk of developing RHD after 3 months of simple anticoagulant therapy was 8.3 times that of the interventional treatment group (95% CI: 0.015–0.994, *P* = 0.049), which may be related to the relatively small sample size in the catheter-directed thrombectomy group. Patients with cardiac structural changes or elevated cardiac biomarkers at admission were more likely to experience adverse events during the follow - up period (17). An increase in RA diameter was associated with RHD (18), which is similar to the results of our study.

In this study, the efficacy of reperfusion therapy was affirmed. However, first of all, a unified definition of RHD has not been established yet. We defined “RHD” based on the normal reference values of clinical echocardiography in our hospital, which may differ from the definition of “RHD” in other literature. Secondly, in this study, a newly - launched domestic thrombectomy device and a low - dose of alteplase for thrombolysis were used, which are different from the drugs and equipment used in previous studies. Finally, this study is a single - center retrospective study with a relatively small sample size, strict inclusion and exclusion criteria, and a short follow - up period. This increases the selection bias and confounding bias. In the future, prospective, multi - center, and large - sample studies are needed to further investigate the impact of reperfusion therapy on the long - term right - heart function and status in patients with intermediate - risk acute pulmonary embolism.

## 5 Conclusion

Early diagnosis and early treatment are the keys to determining the prognosis of APE. Reperfusion therapy can promptly reopen the occluded blood vessels, relieve the burden on the heart and lungs, and improve the hemodynamic status, thereby bringing certain benefits to patients with intermediate-risk PE.

## Data Availability

The raw data supporting the conclusions of this article will be made available by the authors, without undue reservation.
